# RAAAF’s office landscape The End of Sitting: Energy expenditure and temporary comfort when working in non-sitting postures

**DOI:** 10.1371/journal.pone.0187529

**Published:** 2017-11-10

**Authors:** Simone R. Caljouw, Rutger de Vries, Rob Withagen

**Affiliations:** University of Groningen, University Medical Centre Groningen, Center for Human Movement Sciences, A. Deusinglaan 1, Groningen, The Netherlands; University of Illinois at Urbana-Champaign, UNITED STATES

## Abstract

An earlier study suggested that the activity-inviting office landscape called “The End of Sitting”, designed by Rietveld Architecture Art Affordances (RAAAF), should be considered as an alternative working environment to prevent sedentary behavior. The End of Sitting lacks chairs and tables but consists instead of a myriad of sloped surfaces at different heights that afford workers to stand, lean or recline at different locations. In this study, we assessed the impact of four of its workspaces on physical intensity, temporary comfort and productivity of office work and compared the outcomes with sitting and standing behind a desk. Twenty-four participants worked for 10 minutes in each of the six test conditions. Energy expenditure, measured by indirect calorimetry, and heart rate were recorded. Questionnaires were used to assess the perceived comfort. The number of words found in the word search test was counted as a measure of productivity. The majority of The End of Sitting workspaces led to a significant increase in energy expenditure compared with sitting behind a desk (*p*s < .05). Average MET values ranged from 1.40 to 1.58 which is a modest rise in energy expenditure compared to sitting (1.32 METs) and not significantly different from standing (1.47 METs). The scores on the general comfort scale indicated that some workspaces were less comfortable than sitting (*p*s < .05), but the vast majority of participants reported that at least one of The End of Sitting workspaces was equally or more comfortable than sitting. No differences in productivity between the test conditions were found. Further long-term studies are required to assess the behavioral adaptations, productivity and the level of comfort when using The End of Sitting as a permanent office.

## Introduction

**Fig 1:**
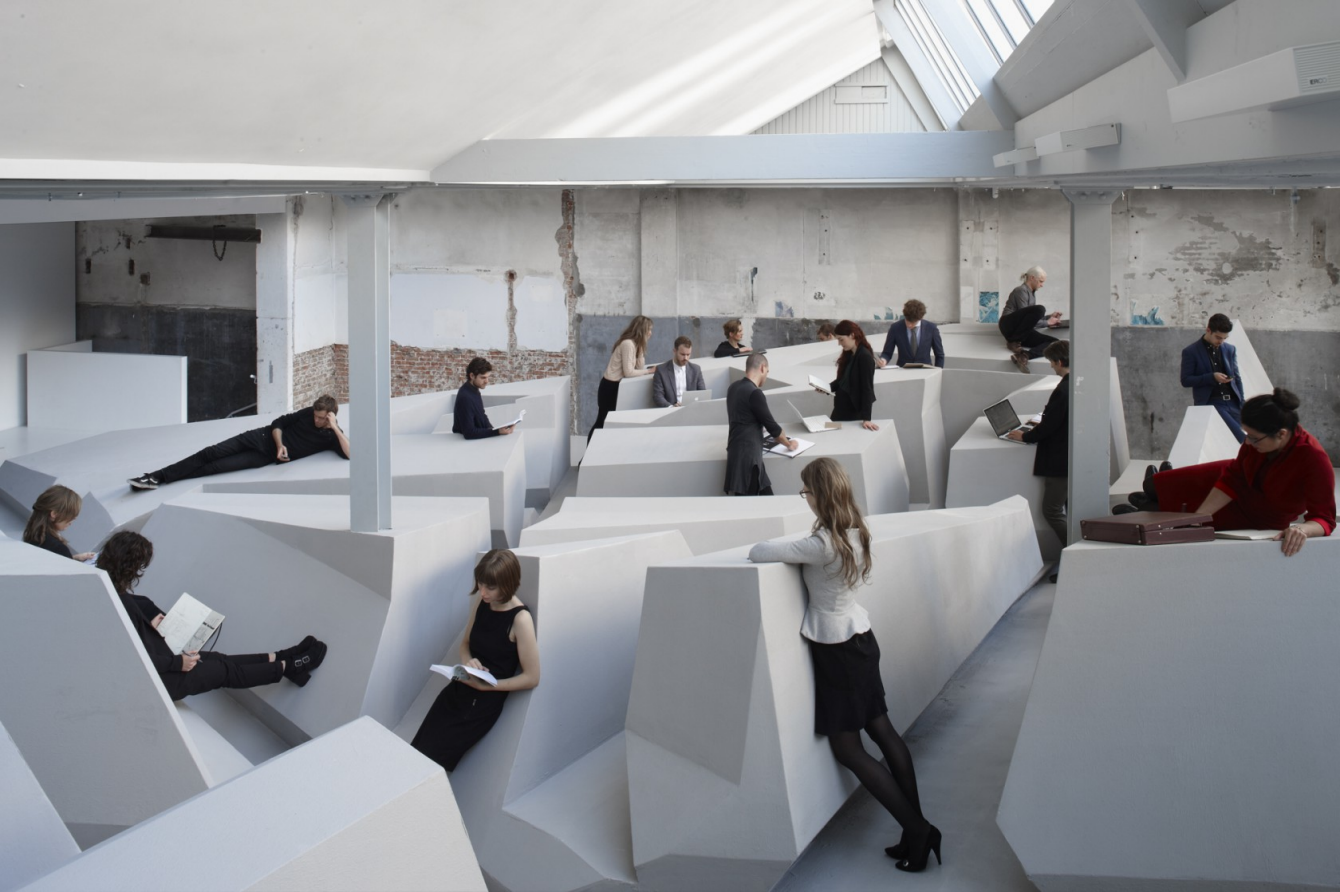
The End of Sitting. The people in this photo did not participate in the study. Reproduced with permission from Kempenaers (available at http://www.raaaf.nl).

People working in an office environment spend on average approximately 10 hours sitting on a work day, often with prolonged periods of sustained sitting [1–5]. Epidemiological evidence associates such prolonged sedentary behavior (i.e. sitting or lying, with lack of muscle contractions and an energy expenditure no greater than 1.5 METs) with an increased risk for a variety of adverse health outcomes [6–8]. Indeed, sedentariness is a risk factor for obesity, type 2 diabetes incidence, cardiovascular disease incidence, cancer incidence, and mortality [1,6,8–11]. Not even regular physical activity can annul the deleterious health consequences of prolonged sitting, although associations become less pronounced as physical activity increases [12–14]. Taking cognizance of this evidence, Rietveld Architecture Art Affordances (RAAAF) and visual artist Barbara Visser opted for a radical change of the working environment and designed a working environment without tables and chairs (see Fig 1). The objective of their art installation called “The End of Sitting” was to create a built environment that invites people to work in a multitude of non-sitting postures [15,16].

Contrary to several activity permissive workstations that are on the market [17–19], RAAAF and Visser intentionally created a myriad of workspaces in a landscape that are comfortable to work in, but not for too long. Hence, they expected people to change posture and/or location in the landscape when they were to work there for a longer period. In an earlier observational study on working in this landscape, Withagen and Caljouw [20] confirmed this hypothesis—83% of the participants worked in different postures at different locations in the landscape. Moreover, compared to working in a conventional office with chairs and tables, the participants reported no negative effects on concentration levels and satisfaction with the produced work. Interestingly, participants felt more energetic after working in The End of Sitting, although their legs felt more tired.

To further develop such landscapes it is important to find out the workspaces in the landscape that invite postures that elevate energy expenditure, offer temporary comfort, and do not reduce productivity. To this end, we selected four workspaces that were frequently used by participants when working in The End of Sitting [20] and compared them with sitting or standing behind a desk. It was expected that working in The End of Sitting workspaces produces a rise in energy expenditure compared to sitting behind a desk A purpose of the design is to provide workspaces that are only temporary comfortable, resulting in workers frequently changing location. Therefore, it was expected that sitting behind a desk is more comfortable than working in The End of Sitting workspaces. Yet, immediate discomfort may prevent the use of certain spaces in the landscape. To evaluate The End of Sitting, we compared the perceived temporary comfort of four of its workspaces with sitting and standing behind a desk. In addition, we also assessed the productivity when working in each of the six conditions.

## Methods

### Participants

Twenty-four participants (10 males, 14 females) between 20 and 27 years of age (mean age 23.1, SD 1.8) volunteered to participate. The height of participants ranged from 1.65 m to 1.98 m (mean height 1.79, SD 0.09), weight ranged from 52.2 kg to 94.3 kg (mean weight 72.5, SD 10.9), and BMI ranged from 18.1 m^2^/kg to 27.5 m^2^/kg (mean BMI 22.6, SD 2.4). All participants were currently enrolled university students. The study was approved by the institutional ethical board of the Center for Human Movement Sciences, University Medical Center Groningen, University of Groningen (ECB/2016.02.15_3), and all participants gave their informed consent.

### Design and procedure

Participants were asked to wear comfortable clothes and flat shoes. They were instructed to refrain from alcohol and exercise for a minimum of 24 h before testing and to refrain from smoking, eating, and drinking fluids with the exception of water for 2 h before testing.

**Fig 2:**
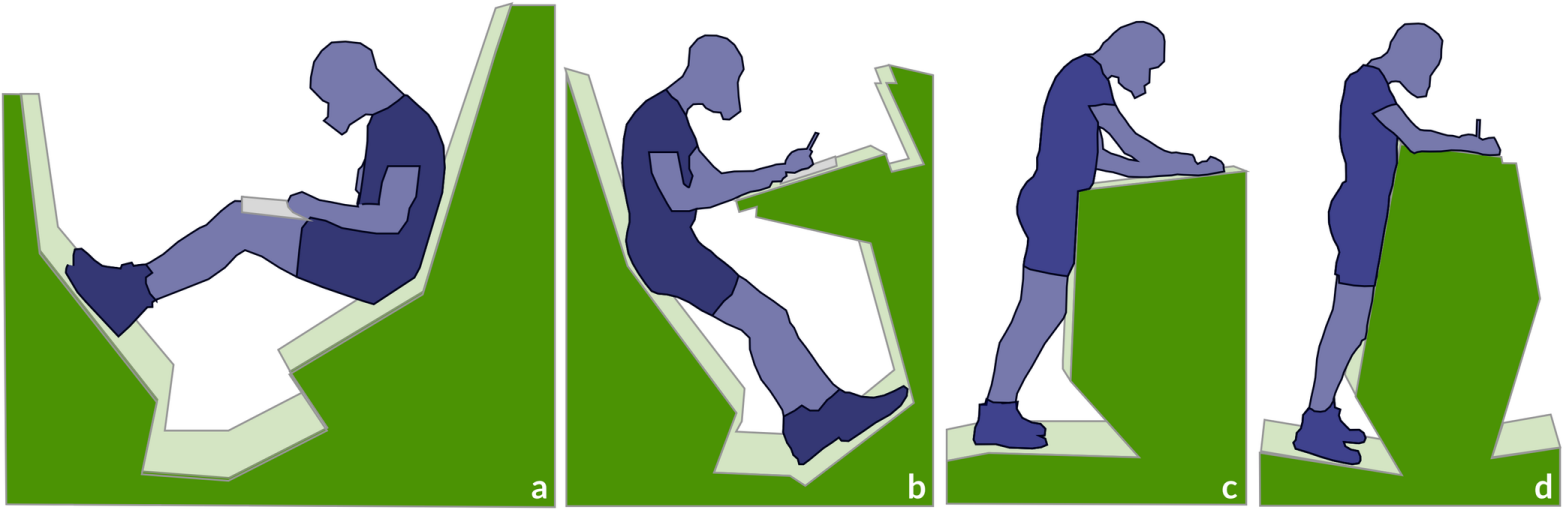
The four The End of Sitting workspaces; (a) Curled up, (b) Lean back, (c) Front low, (d) Front high.

After an initial 15-minute rest period, participants completed six consecutive 10-minute bouts of a word search task in different test conditions: sitting on a chair behind a desk, standing at a desk adjusted to elbow height, and working in the four workspaces selected from The End of Sitting. These workspaces were individual units and were called: Curled up, Lean back, Front low, and Front high (see Fig 2). In each of the six conditions participants were free to make postural alternations (e.g. repositioning on a chair, replacing the feet on the sloped surfaces, turning or bending the torso, extending the arms), but they were not allowed to leave the workspace. Because in the Curled up condition no desk was available, participants used a clipboard when working. All The End of Sitting workspaces were made out of plywood and set-up in the same room with a comfortable temperature of about 23°C and daylight. The tests were conducted using a randomized counterbalanced design. Between test conditions there was a short break of a few minutes and after working in three test conditions there was a mandatory break of 15 minutes in which participants were provided with a glass of water and could rest.

### Measurement instruments and data collection

#### Energy expenditure

Energy expenditure was measured via indirect calorimetry with the Cosmed K4b2 portable gas analyzer (Cosmed, Rome, Italy), allowing almost complete mobility when working in the provided workspaces. The system weighs about 1 kg and is comfortably worn on the chest or on the back (depending on the test condition) using a harness. Gas is collected with a rubber face mask placed over the mouth and nose using a head cap. A room air calibration and gas calibration was carried out before testing and during the break. The Cosmed K4b2 unit includes a Polar T34 chest-strap belt to record heart rate (Polar, Kempele, Finland). The system has been previously validated during rest and light physical activity [21]. The respiratory variables, e.g. oxygen consumption, carbon dioxide production and ventilation, were measured continuously in all test conditions. Data from the final 5 minutes of each 10-minute test period were used to calculate the energy expenditure in a steady state using the formula proposed by Garby & Astrup [22] and the standard METs by dividing *VO*_2_ (ml/min) by 3.5 times the weight of the participant.

#### Task productivity

Productivity was measured with a word search puzzle test [23]. Participants were requested to search and mark as many words as possible. In each test condition participants received a new word search puzzle consisting of about 75 words hidden in a 25 x 17 letter grid that was complex enough to be far from being solved during the 10-minute testing period. The six word search puzzles were of similar difficulty and counterbalanced across test conditions. The number of words found by the participant in each test condition was used as a measure of productivity.

#### Perceived comfort

After each test condition participants were asked to rate the perceived comfort of the workspace on the General Comfort Rating Scale translated to Dutch [24]. The scale is a vertical line of 100 mm with 11 items. Participants draw a horizontal line on the vertical scale to indicate the level of comfort or discomfort. The score was achieved by rounding the mark to the nearest 5 mm point, thereby providing a 0–20 scale. One end of the scale (score 0) reads “I feel completely relaxed”, the middle of the scale (score 10) reads “I feel restless and fidgety”, the other end of the scale (score 20) reads “I feel unbearable pain”. Then a figure of a human body with numbered body parts was presented [25]. Participants identified the most uncomfortable body part by reporting the corresponding number, followed by the second body part with discomfort, and so on, until no other uncomfortable body parts were observed. Afterwards we counted the number of participants who reported discomfort for each of the specified 9 areas (neck, shoulders, arms, upper back, lower back, buttocks, upper legs, lower legs, and feet) per test condition. In addition, we counted the number of areas that each participant reported per condition.

### Statistical analyses

After checking the normality of data, repeated measures ANOVAs with test condition (sitting, standing, Curled up, Lean back, Front low, Front high) as a within-subjects factor were performed to assess differences in energy expenditure, standard METs, heart rate, and number of words found. When the sphericity assumption was violated Huynh-Feldt correction was applied. To further explore the significant effects (p < .05), pairwise comparisons with Bonferroni adjustment were performed to compare The End of Sitting workspaces with the more conventional sitting and standing behind a desk. Friedman tests with test condition as a within-subjects factor were performed to assess the differences in the ratings on the General Comfort Scale, and the reported number of uncomfortable body areas. Wilcoxon signed-rank tests were performed to further assess significant effects and to compare the comfort of The End of Sitting workspaces with sitting and standing behind a desk.

## Results

### Energy expenditure

A significant effect of test condition on energy expenditure (F(5,115) = 9.24, p < .001, $\eta_{p}^{2}$ = 0.29) and METs (F(5,115) = 9.26, p < .001, $\eta_{p}^{2}$ = 0.29) was found. Pairwise comparisons with Bonferroni adjustment indicated that Front high, Front low, and Curled up resulted in a significantly higher energy expenditure and METs than sitting, ps < .05 (see Table 1). Energy expenditure and METs were also significantly higher for standing than for sitting (p = .002). No significant differences in energy expenditure and METs between The End of Sitting workspaces and standing behind a desk were observed. One-sample t-tests were performed to compare the observed MET values per test condition to the traditional MET based threshold for sedentary behavior (<1.5 METs). These tests revealed that only sitting (t(23) = -4,36, p < .001) and Lean back (t(23) = -2.58, p < .05) resulted in an energy expenditure that was significantly lower than the sedentary threshold of 1.5 METs. Energy expenditure in the test conditions standing, Front low, Front high, and Curled up was not significantly different from the 1.5 METs threshold. Looking at the individual differences in METs when working in The End of Sitting workspaces revealed that 13 out of 24 participants exceeded the 1.5 METs threshold in at least two of the four End of Sitting workspaces. Of these 13 participants, 4 participants exceeded the 1.5 METs threshold in *all* The End of Sitting workspaces. For 7 participants energy expenditure was not above the 1.5 METs threshold in any of The End of Sitting workspaces.

**Table 1:** Means (and SDs) of energy expenditure (EEm), metabolic equivalent of task (METs), heart rate, and the number of words found in the word search task for the test conditions; sitting, standing, and the four workspaces from The End of Sitting.

	EEm (kcal/min)	METs	Heart rate (bpm)	Word count
Sitting	1.58 (0.22)	1.32 (0.20)	67.15 (10.10)	22.79 (6.80)
Standing	1.75 (0.33)*	1.47 (0.28)*	75.29 (10.37)*	22.08 (6.45)
Curled up	1.90 (0.43)*	1.58 (0.30)*	69.89 (10.10)	20.88 (5.88)
Lean back	1.67 (0.29)	1.40 (0.20)	72.65 (10.38)*	23.17 (6.98)
Front low	1.75 (0.27)*	1.47 (0.19)*	74.25 (9.85)*	22.04 (8.03)
Front high	1.73 (0.29)*	1.45 (0.22)*	75.60 (11.47)*	21.04 (5.86)

*significantly different from sitting ps < .05

### Heart rate

Mean heart rates and standard deviations for each test condition are also presented in Table 1. These data consist of a sub-collective of 21 out of 24 participants due to technical failure of the heart rate monitor. The repeated measures ANOVA revealed a statistically significant effect of test condition on heart rate (F(5, 100) = 8.29, p < .01, $\eta_{p}^{2}$ = 0.30). Post hoc pairwise comparisons with Bonferroni adjustment revealed that Front high, Front low, and Lean back resulted in higher heart rates than sitting (ps < .05). Interestingly, heart rate in the Curled up workspace was not significantly different from sitting. Heart rate was significantly higher for standing than sitting (p < .001). No significant differences in heart rate were observed between standing and Front high, Front low, and Lean back.

### Task productivity

Table 1 also presents for each test condition the mean number of words found and the standard deviations. The repeated measures ANOVA with test condition (sitting, standing, Curled up, Lean back, Front low, Front high) as the within-subjects factor revealed no significant effect of test condition on the productivity scores (F(5, 115) = 1.16, *p* = 0.34, $\eta_{p}^{2}$ = 0.05).

### Perceived comfort

The Friedman test revealed a significant effect of test condition (χ^2^(5) = 21.84, p < .001) on the general comfort rating. Post hoc Wilcoxon Signed Rank Tests indicated that the perceived comfort ratings of The End of Sitting workspaces were not significantly different from standing (ps>.05). Sitting was perceived as more comfortable than standing (z = 3.06, p < .001), and Curled up (z = 2.40, p < .05) and Front high (z = 2.26, p < .05). The perceived comfort of Front low and Lean back was not significantly different from sitting (ps>.05). A score on the general comfort scale above 10 reflects discomfort. Individual participant data (see Appendix 1) revealed that from the 24 participants two reported scores indicating discomfort when sitting and four when standing behind a desk. In The End of Sitting workspaces, three participants reported discomfort in the Curled up, three in the Lean back, four in the Front low and none in the Front high. Inspection of Table 2 revealed that the interquartile ranges in perceived comfort were smallest for the test conditions sitting and standing, indicating more individual differences in perceived comfort of The End of Sitting workspaces. Looking at the individual differences in perceived comfort of The End of Sitting workspaces, revealed that 19 out of 24 participants perceived at least one workspace as equally or more comfortable than sitting. Of these 19 participants, 2 participants perceived *all* The End of Sitting workspaces as equally or more comfortable than sitting. For only 5 out of the 24 participants, perceived comfort was worse in all The End of Sitting workspaces compared to sitting behind a desk.

**Table 2:** The median and interquartile range of the general comfort rating (scale 0–20: 0 –I feel completely relaxed, 10—I feel restless and fidgety, 20—I feel unbearable pain), the percentage of participants who reported discomfort per body area, and the median number of reported uncomfortable body areas and interquartile range. The asterisks indicate for each body area the test condition with the largest percentage of participants reporting discomfort.

	sitting	standing	Curled up	Lean back	Front low	Front high
general comfort	3.0 (2.0–4.0)	4.5 (4.0–6.0)	5.5 (4.0–8.8)	4.0 (2.0–6.0)	4.0 (2.0–7.3)	4.5 (3.3–6.0)
neck (%)	54	83*	71	33	58	50
shoulders (%)	13	38*	4	13	21	13
arms (%)	4	4	4	13	13	21*
upper back (%)	13	17*	8	4	4	4
lower back (%)	4	17	21	13	13	25*
buttocks (%)	4	0	46*	21	0	0
upper legs (%)	0	0	29*	13	4	4
lower legs (%)	0	8	8	25*	8	13
feet (%)	4	13	17	21*	13	21*
nr. uncomfortable body areas	1.0 (0.3–1.0)	2.0 (1.0–2.8)	2.0 (1.0–2.0)	1.0 (0.0–2.0)	1.0 (0.0–2.0)	1.0 (1.0–2.0)

With regard to the reported number of local body areas with musculoskeletal discomfort, the Friedman test revealed a significant effect of test condition (*χ*^2^(5) = 25.96, p < .001). Post hoc Wilcoxon Signed Rank Tests revealed significantly more body areas with discomfort compared to sitting for the test conditions standing (z = 3.22, p < .001), Lean back (z = 2.04, p < .05), Curled up (z = 3.62, p < .001), and Front high (z = 2.29, p < .05). For this outcome measure no significant differences were observed when comparing the four End of Sitting workspaces with standing (ps > .05). Table 2 shows for each test condition per body area the percentage of participants who reported local discomfort. The neck is frequently reported in all test conditions, including the more conventional conditions (e.g. sitting and standing). Note that this discomfort in the neck might be related to participants wearing a face mask. There is more variation between the test conditions in the other body parts that are reported: the shoulders and back in standing; the buttocks, lower back and upper legs in Curled up; the buttocks, lower legs and feet in Lean back; the shoulders in Front low; and the arms, lower back and feet in Front high.

## Discussion

An earlier study suggested that The End of Sitting should be taken seriously as an alternative working environment that reduces prolonged sitting [20]. The aim of the present study was to examine the energy expenditure of working in four differently designed workspaces provided by The End of Sitting. The results confirmed our expectation that working in most of the tested End of Sitting workspaces produces a modest but significant rise in energy expenditure compared with sitting on a chair behind a desk. It was observed that the workspaces Front high and Front low significantly increased energy expenditure, standard METs, and heart rate compared with sitting. In the Lean back workspace, heart rate significantly increased but energy expenditure was not significantly different from sitting. Contrary, in the Curled up workspace, energy expenditure and METs were higher but heart rate was not significantly different from sitting. Together, these results confirm that the workspaces provided by The End of Sitting office landscape may offer an opportunity to increase the physical intensity of office work.

A major aim of The End of Sitting landscape is to reduce the amount of *sedentary time* at the office [15,16]. Several existing activity-permissive workstations lead to an energy expenditure above 1.5 METs [26,27]. In the present study it was found that most of the tested End of Sitting workspaces elicit an average energy expenditure that is not significantly different from standing behind a desk and not above the 1.5 METs. Activities with such a modest energy expenditure may be defined as sedentary behavior [28] even though the workers do not sit. However, and as mentioned earlier, RAAAF and Visser intentionally designed workspaces that are temporary comfortable and, thus, do not afford working in one workspace for a long time [16], and indeed, the empirical study of Withagen and Caljouw [20] showed that 83% of the participants moved through the landscape and switched between workspaces. Hence, The End of Sitting may reduce sedentary behavior not because of the physical intensity of the specific working postures that are adopted while working in an End of Sitting workspace, but because of the alternation between postures when frequently moving through the landscape from one workspace to another. Future research, involving workers that are accustomed to working in The End of Sitting, should see if workers will keep on moving between the different workspaces throughout a full day of work.

To further develop activity-friendly office landscapes it is important to determine not only the variety of workspaces in the landscape that invite postures that elevate energy expenditure compared to traditional sitting, but also the temporary comfort of these workspaces and the productivity when working in them. No significant differences in word search scores were found between the test conditions, suggesting that The End of Sitting workspaces do not reduce performance. This is in line with the experience of people working longer in The End of Sitting–in the study of Withagen and Caljouw [20] workers reported similar scores on perceived productivity and satisfaction of the performed work in The End of Sitting landscape compared to a traditional office environment. However, the way in which these results are externally valid is unknown. Office work generally covers a wide range of different tasks (typing, moussing, reading, call handling etc.) and difficult to quantify concepts such as creativity, collaboration, and communication can also play a role in productivity.

Contrary to our initial hypothesis, the temporary comfort scores revealed that the Lean back and Front low workspaces were not scored significantly less comfortable than sitting, suggesting that these workspaces might be comfortable alternatives to the traditional chair and desk. Working in the Curled up and Front high workspaces were scored significantly less comfortable than sitting, but not significantly less comfortable than standing. More interestingly, most participants (i.e. 19 out of 24) reported that at least one workspace in The End of Sitting was initially as comfortable as or more comfortable than sitting behind a desk. In general it can be concluded that most of The End of Sitting workspaces do not differ significantly from standing behind a desk in terms of the physical intensity and perceived general comfort. Instead of providing workers with a standing desk, an entire landscape that affords many different postures is an alternative option. The advantage of this variety in workspaces is threefold. First, it invites postural alternations and movement activity to switch from one location to another, because of the temporary comfort of each location. Second, the physical intensity of working in most workspaces offered by the landscape is higher than when sitting behind a desk. Third, working in the different workspaces offers a variation in structural loads. Body areas that are uncomfortable in one workspace are relieved from tension in another workspace.

### Limitations

In the present study, participants worked only for 10 minutes in the different workspaces. Although this gives reliable and valid measures of both energy expenditure and temporary comfort, it leaves us uninformed of the comfort scores taken over longer periods of time, especially regarding the office landscape as a whole (but see Withagen and Caljouw [20]). Moreover, the participants were relatively young and generally physically fit. It is therefore important to further investigate how tolerable The End of Sitting is for older and physically more vulnerable workers.

### Conclusions

The End of Sitting has the potential to increase the energy expenditure of office workers. Most workspaces selected from The End of Sitting were found to increase energy expenditure compared to sitting behind a desk. However, longitudinal studies with a more representative sample of office workers are needed to examine the perceived comfort of working in The End of Sitting, and to evaluate whether this office landscape can serve as a feasible working place of the future as the designers intended.

## Supporting information

S1 Appendix Individual data on energy cost, perceived temporary comfort and productivity for each test condition. (PDF)

S2 Appendix Reported body areas with discomfort for each test condition. (PDF)
