# Supplementary material for: RAAAF’s office landscape The End of Sitting: Energy expenditure and temporary comfort when working in non-sitting postures
Source: PLoS One. 2017 Nov 10;12(11):e0187529. doi: 10.1371/journal.pone.0187529 (PMC5681262; doi:10.1371/journal.pone.0187529)
Supplement: S1 Appendix — (PDF) [file pone.0187529.s001.pdf]

Table 1: Sample characteristics and physical intensity of working in the test conditions

					Physical intensity																	
					Energy expenditure						MET						Heart rate					
Participant	Gender	Age (years)	Weight (kg)	Height (cm)	sitting	standing	Curled up	Lean back	Front low	Front high	sitting	standing	Curled up	Lean back	Front low	Front high	sitting	standing	Curled up	Lean back	Front low	Front high
1.00	male	23.00	83.10	179.00	1.59	1.59	1.90	1.54	1.86	1.67	1.12	1.14	1.37	1.10	1.33	1.19	68.96	76.35	67.12	68.64	67.75	74.84
2.00	female	21.00	75.50	172.50	1.50	1.44	1.45	1.34	1.54	1.62	1.19	1.14	1.15	1.08	1.24	1.31	67.33	81.71	75.08	67.80	70.46	66.32
3.00	female	20.00	57.10	174.50	1.34	1.43	1.49	1.33	1.43	1.43	1.41	1.51	1.57	1.40	1.53	1.51	77.76	81.03	80.48	82.83	79.82	80.16
4.00	male	22.00	87.30	198.00	1.89	2.12	2.65	2.27	2.28	2.22	1.29	1.44	1.79	1.54	1.55	1.52	58.21	67.30	71.09	74.00	72.20	65.05
5.00	male	25.00	85.50	191.00	1.70	1.85	1.99	1.96	2.22	2.16	1.20	1.31	1.40	1.38	1.57	1.53	56.29	66.08	61.11	72.50	82.15	76.85
6.00	male	26.00	80.40	183.00	1.72	1.76	2.39	1.86	1.68	1.66	1.30	1.35	1.82	1.42	1.27	1.26	68.64	71.40	68.20	69.69	71.82	78.89
7.00	male	23.00	63.00	170.50	1.66	1.96	1.72	1.53	1.72	1.97	1.61	1.90	1.66	1.48	1.67	1.91	55.49	59.81	52.64	51.26	54.90	57.42
8.00	male	27.00	72.70	186.50	1.83	2.18	2.27	2.06	2.03	1.92	1.51	1.79	1.87	1.73	1.70	1.60	84.57	86.77	86.01	81.21	84.79	80.24
9.00	female	23.00	52.20	170.00	1.67	1.75	2.08	1.51	1.69	1.53	1.96	2.05	2.44	1.77	1.99	1.79	70.92	86.10	61.98	79.74	78.02	77.61
10.00	female	25.00	66.30	175.00	1.42	1.54	1.65	1.58	1.55	1.60	1.29	1.39	1.49	1.43	1.41	1.46	61.70	70.91	69.10	72.98	73.06	67.79
11.00	male	21.00	74.10	185.00	1.99	2.50	2.25	1.98	2.22	2.15	1.61	2.03	1.82	1.59	1.78	1.72						
12.00	female	23.00	82.50	183.00	1.72	1.55	2.61	1.55	1.81	1.53	1.23	1.12	1.88	1.13	1.31	1.11	87.52	94.43	87.56	97.68	90.84	99.90
13.00	female	23.00	62.30	175.00	1.22	1.43	1.17	1.44	1.68	1.56	1.18	1.38	1.14	1.40	1.64	1.52	75.09	86.72	81.52	76.76	80.58	80.49
14.00	male	22.00	94.30	192.00	1.77	2.35	2.12	2.07	1.96	1.84	1.15	1.52	1.37	1.35	1.28	1.19	56.58	75.38	69.26	71.10	63.84	62.59
15.00	female	22.00	73.90	180.00	1.62	1.76	2.00	1.57	1.89	1.82	1.34	1.46	1.65	1.29	1.56	1.50						
16.00	female	23.00	78.60	169.00	1.91	2.05	2.34	2.21	1.91	2.09	1.46	1.59	1.81	1.70	1.46	1.61	63.83	68.52	63.89	76.31	87.88	81.62
17.00	female	24.00	74.50	174.00	1.50	1.45	1.58	1.45	1.59	1.66	1.19	1.17	1.27	1.17	1.27	1.32	52.86	51.76	51.06	53.26	59.83	62.14
18.00	female	23.00	64.70	167.00	1.38	1.35	1.62	1.45	1.45	1.40	1.28	1.28	1.51	1.36	1.36	1.31	57.36	66.77	56.87	56.62	61.44	64.82
19.00	female	24.00	66.80	178.00	1.48	1.75	1.56	1.33	1.41	1.37	1.32	1.58	1.40	1.20	1.28	1.24	70.24	87.39	76.71	66.53	69.41	74.32
20.00	male	27.00	78.90	192.00	1.44	1.57	1.99	1.57	1.63	1.53	1.09	1.19	1.50	1.19	1.24	1.16	57.07	75.38	70.71	74.53	65.20	66.59
21.00	female	22.00	66.80	165.00	1.23	1.30	1.42	1.80	1.53	1.39	1.07	1.13	1.22	1.55	1.32	1.21						
22.00	female	21.00	54.10	167.00	1.24	1.44	1.28	1.32	1.39	1.42	1.36	1.60	1.41	1.46	1.53	1.57	64.64	68.08	64.87	74.65	79.41	82.82
23.00	male	22.00	81.10	186.00	1.62	2.00	2.49	1.74	1.99	2.32	1.18	1.48	1.83	1.28	1.46	1.70	76.03	79.95	72.29	76.94	85.57	100.12
24.00	female	23.00	63.30	174.00	1.46	1.86	1.49	1.61	1.53	1.68	1.38	1.79	1.43	1.54	1.46	1.59	79.12	79.31	80.13	80.55	80.30	87.02

Table 2. Perceived temporary comfort and productivity when working in the test conditions

Participant	Temporary comfort												Productivity					
	General Comfort Score (scale 0-20)						number of uncomfortable body areas						number of words found					
	sitting	standing	Curled up	Lean back	Front low	Front high	sitting	standing	Curled up	Lean back	Front low	Front high	sitting	standing	Curled up	Lean back	Front low	Front high
1	3	4	4	3	3	4	1	4	5	6	4	5	24	27	24	36	29	25
2	2	6	4	2	0	2	0	1	1	0	0	1	36	16	26	28	33	23
3	5	13	5	2	13	3	2	2	2	1	2	2	18	14	11	14	10	12
4	2	6	8	7	4	4	3	5	5	4	5	3	22	28	23	20	24	28
5	3	4	12	4	10	4	0	0	2	0	1	1	13	17	14	13	9	15
6	13	7	4	4	8	5	2	2	1	1	2	1	28	22	22	19	23	20
7	2	3	3	3	2	3	0	1	1	1	1	1	12	19	19	24	25	17
8	2	4	4	2	2	2	0	1	1	0	0	0	31	18	22	33	26	23
9	5	13	9	5	14	7	1	1	3	2	2	2	19	13	12	18	11	10
10	2	6	8	4	2	8	1	3	2	0	0	1	31	29	22	29	27	21
11	4	4	6	8	4	6	1	3	2	2	0	1	24	31	30	22	15	27
12	5	6	12	6	5	5	1	1	4	3	2	2	28	26	25	33	28	28
13	3	3	1	2	1	4	1	2	2	1	1	1	30	25	30	25	34	30
14	3	11	9	16	5	8	1	2	3	3	2	2	13	18	12	15	15	15
15	14	13	9	3	5	2	1	3	2	1	1	0	26	26	20	14	22	21
16	4	4	0	6	10	6	2	3	1	2	0	2	12	22	20	20	10	21
17	4	4	3	2	2	6	1	1	1	1	1	1	32	35	32	33	38	27
18	0	6	14	12	4	6	0	2	2	2	1	1	26	32	27	32	31	26
19	2	5	4	4	2	3	1	2	2	2	1	1	17	10	19	18	22	25
20	3	3	4	3	5	5	1	0	2	2	1	1	21	26	14	22	22	23
21	2	4	8	12	14	4	1	2	2	3	4	3	19	19	18	16	16	16
22	3	4	6	4	3	7	0	0	1	0	0	2	18	20	15	23	20	26
23	3	4	8	2	4	5	1	1	2	0	1	2	23	16	21	20	17	12
24	2	6	3	2	2	4	1	1	1	0	0	0	24	21	23	29	22	14
