# Supplementary material for: RAAAF’s office landscape The End of Sitting: Energy expenditure and temporary comfort when working in non-sitting postures
Source: PLoS One. 2017 Nov 10;12(11):e0187529. doi: 10.1371/journal.pone.0187529 (PMC5681262; doi:10.1371/journal.pone.0187529)
Supplement: S2 Appendix — (PDF) [file pone.0187529.s002.pdf]

Table 3. Body areas with discomfort in each test condition

Participant	sitting								
	Neck	Shoulders	Arms	Upper back	Lower back	Buttocks	Upper legs	Lower legs	Feet
1	0	0	0	1	0	0	0	0	0
2	0	0	0	0	0	0	0	0	0
3	1	0	0	0	0	0	0	0	1
4	0	1	1	0	0	1	0	0	0
5	0	0	0	0	0	0	0	0	0
6	1	1	0	0	0	0	0	0	0
7	0	0	0	0	0	0	0	0	0
8	0	0	0	0	0	0	0	0	0
9	1	0	0	0	0	0	0	0	0
10	1	0	0	0	0	0	0	0	0
11	1	0	0	0	0	0	0	0	0
12	0	0	0	0	1	0	0	0	0
13	1	0	0	0	0	0	0	0	0
14	1	0	0	0	0	0	0	0	0
15	1	0	0	0	0	0	0	0	0
16	1	1	0	0	0	0	0	0	0
17	1	0	0	0	0	0	0	0	0
18	0	0	0	0	0	0	0	0	0
19	1	0	0	0	0	0	0	0	0
20	0	0	0	1	0	0	0	0	0
21	1	0	0	0	0	0	0	0	0
22	0	0	0	0	0	0	0	0	0
23	1	0	0	0	0	0	0	0	0
24	0	0	0	1	0	0	0	0	0

Participant	standing								
	Neck	Shoulders	Arms	Upper back	Lower back	Buttocks	Upper legs	Lower legs	Feet
1	1	1	0	1	0	0	0	0	1
2	1	0	0	0	0	0	0	0	0
3	1	1	0	0	0	0	0	0	0
4	1	1	1	0	1	0	0	0	1
5	0	0	0	0	0	0	0	0	0
6	1	1	0	0	0	0	0	0	0
7	1	0	0	0	0	0	0	0	0
8	1	0	0	0	0	0	0	0	0
9	1	0	0	0	0	0	0	0	0
10	1	0	0	1	0	0	0	0	1
11	1	0	0	1	1	0	0	0	0
12	1	0	0	0	0	0	0	0	0
13	1	0	0	0	0	0	0	1	0
14	1	1	0	0	0	0	0	0	0
15	1	1	0	1	0	0	0	0	0
16	1	1	0	0	0	0	0	1	0
17	1	0	0	0	0	0	0	0	0
18	1	0	0	0	1	0	0	0	0
19	1	1	0	0	0	0	0	0	0
20	0	0	0	0	0	0	0	0	0
21	1	0	0	0	1	0	0	0	0
22	0	0	0	0	0	0	0	0	0
23	0	1	0	0	0	0	0	0	0
24	1	0	0	0	0	0	0	0	0

Participant	Lean back								
	Neck	Shoulders	Arms	Upper back	Lower back	Buttocks	Upper legs	Lower legs	Feet
1	1	0	1	0	0	1	1	1	1
2	0	0	0	0	0	0	0	0	0
3	0	0	1	0	0	0	0	0	0
4	1	1	0	0	1	0	0	0	1
5	0	0	0	0	0	0	0	0	0
6	0	1	0	0	0	0	0	0	0
7	1	0	0	0	0	0	0	0	0
8	0	0	0	0	0	0	0	0	0
9	1	0	0	0	0	0	1	0	0
10	0	0	0	0	0	0	0	0	0
11	0	1	0	0	0	0	0	1	0
12	0	0	0	0	1	1	0	1	0
13	0	0	0	0	0	0	0	1	0
14	0	0	1	0	0	1	0	0	1
15	1	0	0	0	0	0	0	0	0
16	0	0	0	0	0	1	0	1	0
17	1	0	0	0	0	0	0	0	0
18	0	0	0	0	1	0	0	0	1
19	1	0	0	0	0	1	0	0	0
20	1	0	0	0	0	0	0	1	0
21	0	0	0	1	0	0	1	0	1
22	0	0	0	0	0	0	0	0	0
23	0	0	0	0	0	0	0	0	0
24	0	0	0	0	0	0	0	0	0

Participant	Curled up								
	Neck	Shoulders	Arms	Upper back	Lower back	Buttocks	Upper legs	Lower legs	Feet
1	1	0	0	0	1	1	1	0	1
2	0	0	0	0	1	0	0	0	0
3	1	0	0	0	0	1	0	0	0
4	1	0	1	0	1	1	1	0	0
5	0	0	0	0	0	1	1	0	0
6	0	0	0	0	0	0	1	0	0
7	1	0	0	0	0	0	0	0	0
8	1	0	0	0	0	0	0	0	0
9	1	1	0	0	0	0	1	0	0
10	0	0	0	1	0	0	0	0	1
11	1	0	0	1	0	0	0	0	0
12	0	0	0	0	0	1	1	1	1
13	1	0	0	0	0	1	0	0	0
14	1	0	0	0	0	1	0	1	0
15	1	0	0	0	0	0	0	0	1
16	0	0	0	0	1	0	0	0	0
17	1	0	0	0	0	0	0	0	0
18	0	0	0	0	1	1	0	0	0
19	1	0	0	0	0	1	0	0	0
20	1	0	0	0	0	0	1	0	0
21	1	0	0	0	0	1	0	0	0
22	1	0	0	0	0	0	0	0	0
23	1	0	0	0	0	1	0	0	0
24	1	0	0	0	0	0	0	0	0

Participant	Front low								
	Neck	Shoulders	Arms	Upper back	Lower back	Buttocks	Upper legs	Lower legs	Feet
1	1	0	1	0	0	0	0	1	1
2	0	0	0	0	0	0	0	0	0
3	0	0	0	0	0	0	0	1	1
4	1	1	1	0	1	0	0	0	1
5	1	0	0	0	0	0	0	0	0
6	1	1	0	0	0	0	0	0	0
7	1	0	0	0	0	0	0	0	0
8	0	0	0	0	0	0	0	0	0
9	1	1	0	0	0	0	0	0	0
10	0	0	0	0	0	0	0	0	0
11	0	0	0	0	0	0	0	0	0
12	1	1	0	0	0	0	0	0	0
13	1	0	0	0	0	0	0	0	0
14	1	0	1	0	0	0	0	0	0
15	0	1	0	0	0	0	0	0	0
16	0	0	0	0	0	0	0	0	0
17	1	0	0	0	0	0	0	0	0
18	0	0	0	0	1	0	0	0	0
19	1	0	0	0	0	0	0	0	0
20	1	0	0	0	0	0	0	0	0
21	1	0	0	1	1	0	1	0	0
22	0	0	0	0	0	0	0	0	0
23	1	0	0	0	0	0	0	0	0
24	0	0	0	0	0	0	0	0	0

Participant	Front high								
	Neck	Shoulders	Arms	Upper back	Lower back	Buttocks	Upper legs	Lower legs	Feet
1	1	0	1	1	1	0	0	0	1
2	0	0	0	0	1	0	0	0	0
3	0	0	1	0	0	0	0	1	0
4	0	1	1	0	0	0	0	0	1
5	1	0	0	0	0	0	0	0	0
6	0	0	0	0	0	0	0	1	0
7	1	0	0	0	0	0	0	0	0
8	0	0	0	0	0	0	0	0	0
9	1	0	0	0	1	0	0	0	0
10	0	0	1	0	0	0	0	0	0
11	1	0	0	0	0	0	0	0	0
12	1	1	0	0	0	0	0	0	0
13	1	0	0	0	0	0	0	0	0
14	1	0	1	0	0	0	0	0	0
15	0	0	0	0	0	0	0	0	0
16	0	0	0	0	0	0	0	1	1
17	1	0	0	0	0	0	0	0	0
18	0	1	0	0	0	0	0	0	0
19	1	0	0	0	0	0	0	0	0
20	0	0	0	0	1	0	0	0	0
21	1	0	0	0	1	0	1	0	0
22	0	0	0	0	1	0	0	0	1
23	1	0	0	0	0	0	0	0	1
24	0	0	0	0	0	0	0	0	0
